# Can Microplastics Influence the Accumulation of Pb in Tissues of Blue Crab?

**DOI:** 10.3390/ijerph18073599

**Published:** 2021-03-30

**Authors:** Paula Munuera, Inmaculada Salvat-Leal, Antonio Belmonte, Diego Romero

**Affiliations:** 1 Área de Toxicología, Campus de Espinardo, Universidad de Murcia, 30100 Murcia, Spain; paulamunueraformigo@gmail.com (P.M.); inmaculada.salvat@um.es (I.S.-L.); 2 Taxon Estudios Ambientales, C/Uruguay (Pol I), s/n–Par. 8/27. Nave 31, 30820 Murcia, Spain; antonio.belmonte@taxon.es

**Keywords:** blue crab, gills, hepatopancreas, lead, meat, microplastics

## Abstract

The study of microplastics (MPs) and associated pollutants is essential for a better understanding of some of the factors that threaten marine ecosystems. The main objective of this study was thus to assess Pb distribution and accumulation in the tissues of blue crabs (*Callinectes sapidus*) exposed to MPs. Blue crabs were collected from the mouth of the river Segura (Guardamar, Spain) and fed on mussels from two Mediterranean areas with different levels of Pb contamination: Portmán Bay and San Pedro del Pinatar (Murcia, Spain). In addition, a batch of each group were exposed to MPs. After seven days of exposure, the crabs were euthanised, and tissues and faeces were analysed. The hepatopancreas was found to be the best tissue for measuring Pb concentrations after feeding; muscle tissue did not provide information on environmental quality. The meat (muscle) consumption of blue crabs from zones with high Pb content does not seem to constitute a risk for consumers, although the risk is not totally negated if all soft tissues are ingested. The presence of MPs in the water does not seem to increase the accumulation of Pb in these tissues of *C. sapidus*.

## 1. Introduction

In the twenty-first century, marine pollution is now treated as a serious problem by scientists, governments, and society alike. The presence of pollutants in the sea poses a threat to wildlife, human beings, and ecosystems, even though its impact is very hard to quantify. Marine ecosystems have to face up to a cocktail of chemical pollutants. In addition to inherited contaminants such as heavy metals, there are a series of substances such as plastics [1,2,3] whose impact not only negatively affects marine species but could also be a threat to human health via the food chain.

Heavy metals represent one of the most serious pollution concerns in marine ecosystems [4,5] and, due to their toxic character, long-term persistence, and ability to enter and bioaccumulate in food webs, they constitute an important threat to human beings and aquatic life [6]. Lead is a non-essential, widely used toxic metal that has generated pollution and health problems worldwide [7]. It is regarded as a priority toxicant by the US Environmental Protection Agency [8] and Spanish legislation [9]. The presence of this metal in the environment continues to cause concern [10] and more guidelines are required to control which species of fish and seafood are consumed by humans, and how often, regarding the state of environmental pollution [11].

Waste plastic is a further concern because it is present in marine and freshwater ecosystems all over the world to such an extent that today plastic litter constitutes about 85% of all marine litter; its worldwide production has risen significantly and in 2018, 359 million tonnes of plastic were produced [12]. According to Jambeck et al. [13], by 2025, it is thought that 50–250 million tons of marine contamination will have originated from land-based sources. Larger plastic remnants break up into smaller pieces, giving rise to ‘microplastics’ (MPs), that is, fragments measuring <5 mm [14,15]. The MPs that have entered the sea remain there and are transported by winds and surface currents to different parts of the world in the form of water bodies [16,17]. This proliferation of MPs has led to the emergence of threats related to wildlife and human health. Interactions between heavy metals and MPs have been recorded, and MPs are thought to potentially act as vectors for heavy metals, thereby increasing the exposure of living organisms to these pollutants [18].

In light of the above, environmental monitoring plans in which aquatic organisms are used as ‘sentinel’ or ‘bioindicators’ are employed to evaluate the presence of plastics and heavy metals in marine ecosystems [19]. The blue crab (*Callinectes sapidus*) and the mussel (*Mytilus* spp.) are common species that are often used for this purpose [20,21,22]. The blue crab is a swimming decapod from the western coast of the Atlantic and the Caribbean, and it is widely distributed [23,24]. It is regarded as a euryhaline species because of its capacity to hyperosmoregulate in the salinities in which it lives [25]. Its distribution is influenced by the temperature and salinity of the water, being commonest in estuaries and seas with muddy bottoms [26,27,28]. In 2016, the Spanish Ministry of Fisheries included *C. sapidus* on its list of commercial fish species [29], and it is nutritionally well-accepted when described as ‘Mediterranean blue crab meat’ [30,31,32]. Studies of metal contamination in this species have multiple objectives, including its effects on human health and the fitness of crabs themselves [33], given that they reside in surface sediments and feed on benthic prey that normally live in contaminated areas. Furthermore, the crabs do not require authorisation for experimental procedures, and capture techniques do not involve any potential risks from an environmental point of view. Bivalves are by far the most widely used pollutant indicator organisms in environmental control studies in coastal areas [34]. Mussels are commonly used indicator species due to their wide distribution, abundance in the wild, and easy manipulation. Moreover, due to their well-documented usefulness and effectiveness, they are still extensively used for biomonitoring and experimental procedures (e.g., [35,36,37,38,39]). Their sessile condition and benthic and sedentary nature ensure that they persist in the area in which they are placed even if the environment is polluted [40]. Additionally, their capacity to filter [41] favours the entrance of contaminants into their systems, thereby allowing for the accumulation of chemical substances in their tissues whose analysis can provide key information about environmental pollution levels [42]. In this context, the aim of the present study was to assess how MPs affect lead concentrations in the tissues of *C. sapidus* fed on mussels taken from two Mediterranean environments with differing levels of Pb pollution.

## 2. Materials and Methods

### 2.1. Sample Collection and Conditioning Period

#### 2.1.1. Mussels

The mussels (*Mytilus galloprovincialius*) used in this study (4–5 cm in length) were obtained from an aquaculture farm located in the Ebro delta (Spain) in October 2018. Once their initial Pb concentrations had been analysed, mussels were relocated in two areas: (1) Portmán Bay, one of the areas with the highest levels of metal pollution in the whole Mediterranean basin [40,43,44], and (2) San Pedro del Pinatar, a non-polluted area [45]. For each location, three sampling stations were set up (coordinates: 689492X/4161139Y, 689559X/4161080Y, 689710X/4161065Y for Portmán Bay; and 704427X/4186597Y, 704387X/4186604Y, 704340X/4186601Y for San Pedro del Pinatar), and in each station, three bags, each of 30 mussels, were placed. After 113 days, the bags were retrieved: one half of the mussels from each bag was used to perform the analysis of the Pb concentrations and the other half to feed the crabs. Mussels were stored at −20° until the experiment began.

#### 2.1.2. Blue Crabs

Thirty blue crabs were collected from the mouth of the river Segura (RSM) (Alicante province, coordinates 705558X-4220269Y) in October 2019 with a fyke net. The net was placed on the bottom of the river and retrieved four hours later. Crabs were brought back to the laboratory in containers filled with water, and 24 were acclimatised for 10 days in tanks of artificial seawater (ASW) under laboratory conditions. The salinity of the ASW was similar to that of the RSM (osmolarity of 94.7 ± 2.3 mmol kg^−1^). The other experimental conditions were as follows: pH = 8.1 ± 0.04, temperature = 18.0 ± 1.1 °C, continuous aeration, and a natural photoperiod. During their acclimatisation, crabs were fed on mussels from the Ebro delta (Spain) at a food equivalent of 4–5% of their weight and a Pb concentration of 0.070 ± 0.006 mg Kg^−1^ (wet weight). The biometric measures (geometric mean ± standard error) of crabs were 45.1 ± 2.8 g (weight), 4.3 ± 0.1 cm (carapace length between abdominal segment and rostrum) and 8.9 ± 0.3 cm (carapace width between lateral spines). The remaining crabs (*n* = 6, the ‘Zero Hour’, unexposed group) were euthanised immediately by hypothermia (30–40 min at −20 °C), sexed and their biometric parameters (weight, length and width) were recorded. Tissues from each specimen (hepatopancreas, muscle and gill) were carefully removed from the carapace with a surgical knife, transferred to 1.5-mL microtubes and stored at −20° until processed.

### 2.2. Experimental Procedure

**Figure 1:**
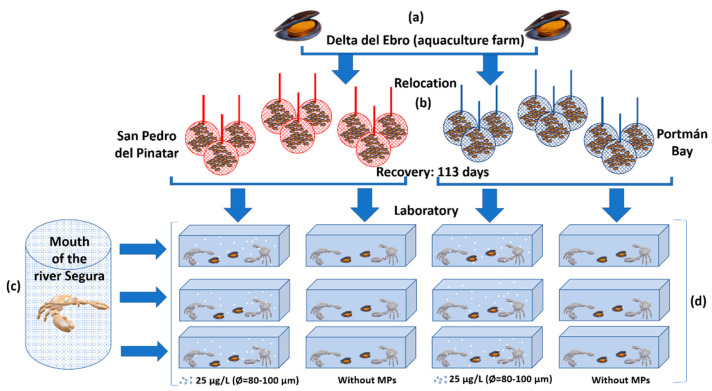
Phases of the study: (**a**) mussel acquisition (aquaculture farm, Delta del Ebro, Spain), (**b**) mussel relocation (San Pedro del Pinatar and Portmán Bay, Spain), (**c**) crab collection (mouth of the river Segura, Spain), and (**d**) experimental procedure (crabs fed on mussels from San Pedro del Pinatar or Portmán Bay, with or without microplastics (MPs) in the water).

Four groups were prepared based on feeding and MP exposure (Figure 1): (1) blue crabs fed on mussels with high Pb concentration from Portmán Bay with (1.1) or without (1.2) MPs in the water; and (2) blue crabs fed on mussels with low Pb concentrations from San Pedro del Pinatar with (2.1) or without (2.2) MPs in the water. The daily diet and consumption of each crab was registered and was equal to 5.5 ± 0.20% of their body weight (geometric mean ± standard error). For both groups, the MPs used were Aquatex−100, an oxidised polyethylene with a particle size of 80–100 µm and 0.99 g cc^−1^ density at 25 °C. The MP concentration was 25 µg L^−1^, a realistic concentration that corresponds to the studies performed by Green et al. [46] and matches the prediction by Jambeck et al. [13] regarding the increase of global plastic waste by 2025. The experiment was carried out in three tanks per treatment (eight litres of ASW per tank, with the same experimental conditions of pH, osmolarity, temperature, aeration, and photoperiod, as described above) with two crabs in each tank (*n* = 6 crabs per treatment) for seven days. Once the experiment concluded, crabs were euthanised, and tissues were removed as described for crabs from the ‘Zero Hour’ group. As well, faeces were collected daily and placed in different tubes for each tank, then rinsed three times with bidistilled water, centrifuged, and dried at +60 °C until constant weight. Faeces from each group were pooled due to the low weight of the collected faeces. In addition, pools of several days were grouped: days 1–2, 3–4–5, and 6–7.

### 2.3. Pb Analysis

To determine their Pb content, tissue and faeces samples, and ASW were analysed using inductively coupled plasma optical emission spectrometry (ICP-OES, ICAP 6500 Duo, Thermo Scientific, USA, with One Fast System, USA). Hepatopancreas, gill, and muscle tissues, as well as faeces, were treated with trace mineral grade nitric acid (69% Suprapure, Merck) and 33% H2O2 (Suprapure, Merck) in special Teflon reaction tubes. These tubes were heated at 220 °C for two minutes in a microwave digestion system (UltraClave-Microwave Milestone^®^, Italy) and later diluted to 10 mL with double deionised water (Milli-Q). The limit of detection was 0.001µg g^−1^. Two replicates were analysed for every sample; the concentration values used in the analysis were the mean of two readings. For every 11 samples, one blank sample was analysed in the ICP-OES to check for possible metal contamination. Taking UNE-EN ISO 11885 as a reference for the determination of elements by ICP atomic emission spectroscopy, multi-element calibration standards (SCP Science, in 4% nitric acid) were prepared with specific Pb concentrations. Intermediate patterns were prepared for this element. The calibration device was established per batch, with a minimum of three points for every lot. Each run started with the calibration standards, continued with samples and intermediate patterns, and finished with the series with intermediate patterns (10% variation coefficient). The wavelength was 220.353 nm. The uncertainty and recovery percentages were 6.14 and 96.44, respectively, and the standard reference material was L577b (bovine liver). Lead concentrations were expressed in micrograms per gram in wet weight for tissues (μg g^−1^ ww) and dry wet for faeces (μg g^−1^ dw).

### 2.4. Data Analysis

The data are presented as geometric means ± standard errors of the means, and minimum and maximum concentrations. According to international guidelines [47], the middle-bound approach was taken (i.e., the values below the detection limit were equal to half this limit) to calculate mean concentrations. Lead concentrations were compared between exposure groups (by tissue and faeces) and between tissues (by exposure group). To check for data normality, a Shapiro-Wilk test was used. For the study of the groups, a log transformation of the data was carried out and parametric mean comparison tests were conducted (Student *t*-test and ANOVA with Tukey and Games Howell post-hoc test, and Levene’s test for the equality of variances). Marginal differences between groups (*p* = 0.05–0.1) were confirmed by a non-parametric test (U de Mann–Whitney). Spearman’s rank correlation coefficient test was applied (a) to Pb concentrations between tissues from each group, and (b) to biometric data and Pb concentrations from each tissue and exposure group. To compare the biometric measures, a Pearson test was carried out. In all cases, *p*-values of less than 0.05 were taken to be statistically significant. All statistical analyses were performed with IBM SPSS Statistics v.19.0 (IBM, New York, NY, USA) for Windows.

## 3. Results and Discussion

The Pb concentrations detected in mussels from the aquaculture farm in the Ebro delta were 0.076 mg kg^−1^, while in mussels collected after the experimental period from San Pedro del Pinatar and Portmán Bay, concentrations were 0.220 ± 0.009 and 3.652 ± 0.188 mg kg^−1^, respectively. These concentrations are realistic and are similar to others reported in previous studies [48]. The relationship of the Pb concentrations between the two ecosystems (unpolluted vs. polluted, 1:17) differed from those reported by other authors (1:2 [22]; 1:4 [49]). Based on the ingested food quantity and Pb concentrations, we estimated that the total Pb consumed by each crab (geometric mean ± standard error) was 23.5 ± 1.0 µg (for crabs fed on mussels from San Pedro del Pinatar) and 529.2 ± 20.4 µg (for crabs fed on mussels from Portmán Bay). Moreover, there was a close relationship (r > 0.8, *p* < 0.001) between the µg of ingested Pb and the biometric data (weight, carapace length, and carapace width) in both groups (crabs fed on mussels from San Pedro del Pinatar and from Portmán Bay), which guaranteed that the Pb ingested was proportional to the size of the crabs.

Descriptive data of Pb concentrations in the crab tissues are given in Table 1. San Pedro del Pinatar is considered by environmental studies to be a non-polluted area [45] and so, as expected, the crabs fed on mussels from this area had low Pb concentrations in their tissues. These concentrations were lower than those reported for the same species in other Mediterranean areas such as the Köyceğiz Lagoon (Turkey), Acquatina Lagoon (Italy), İskenderun Bay (Turkey) and Mersin Bay (Turkey) [20,31,50,51,52], and also lower than from countries such as USA, Brazil, and Venezuela [33,53,54,55,56]. The non-exposed crabs (‘Zero Hour’ group) had the same Pb concentration as those fed on mussels from San Pedro del Pinatar, which could be due to the fact that, after winning the European Riverprize Awards in 2016, the river Segura is now one of the least polluted rivers in Spain. On the other hand, Portmán Bay is one of the most polluted areas in the Mediterranean basin [40,43,44] and in the tissues of crabs fed on mussels from this latter area, Pb concentrations were higher than those reported by Genç and Yilmaz [20], but lower than those reported by a number of other authors [31,50,51,52]. In terms of countries such as USA, Brazil and Venezuela, the results were lower [33,54] or higher [53,55,56], depending on the tissue that was analysed.

The order of Pb concentrations was the following (the same superscript indicates significant statistical differences between tissues): hepatopancreas^1^ > gills^2^ > muscle^1,2^ in crabs from the control group (‘Zero Hour’) and crabs fed on mussels from San Pedro del Pinatar; and hepatopancreas^1,2^ > gills^1,3^ > muscle^2,3^ in crabs fed on mussels from Portmán Bay. According to Duruibe et al. [57], the accumulation of heavy metals is higher in chest tissues than in appendages, and muscles only accumulate a small amount of metal [58], which agrees with our results. In this sense, a low percentage of crabs with Pb above DL in muscles was recorded (56.7%), which coincides with the figures reported by Sivaperumal et al. [59], while most of the hepatopancreas and gill samples had Pb concentrations above the DL (96.7%).

Both these tissues (hepatopancreas and gill) commonly have the highest amounts of metal due to their nature and the role they play in the organism. The hepatopancreas is the principal tissue for storing toxicants, while the gills are a large absorptive organ system and one of the main points in which crabs are in contact with the noxious substances [24]. Regarding metals, the hepatopancreas (digestive gland) has been described as the main organ for detoxification and accumulates more toxicants than the gills [20,60]. However, no significant differences were reported between these two tissues in the crabs from the ‘Zero Hour’ group and the crabs fed on mussels from San Pedro del Pinatar (Table 1). It is known that metals can enter the organism in two ways [54,61,62]: (1) directly via the water that enters the gills or (2) indirectly via diet, which could explain the differences found between the hepatopancreas and the gills in the crabs fed on mussels from Portmán Bay given that the metal concentrations in the water in the tanks were low (0.007 ± 0.008 mg L^−1^).

Interestingly, when these organs reach saturation, it is thought metal excess is transferred to other tissues via hemolymph [24,49]. Larger crabs were fed on greater amounts of food to ensure that they ingested more total Pb (r > 0.8, *p* < 0.05). However, there was no correlation between the biometric data and Pb concentrations in tissues. On the other hand, the concentration of Pb in faeces was almost 20 times higher in individuals fed on mussels from Portmán Bay than in individuals fed on mussels from San Pedro del Pinatar (Table 1). Although faeces were collected daily, we cannot confirm whether the crabs from both exposure groups excreted Pb on a daily basis or whether Pb was concentrated in the most recent excretions. However, this efficiency in Pb excretion through faeces could help avoid a generalised spread throughout the animal and so explain why no correlation was detected between the biometric data and Pb concentrations in tissues. Metal uptake and its regulation in invertebrates have been explained by theoretical models. Rainbow [63] has stated that crustaceans could assimilate non-essential metals (1) without excreting them and store them in a detoxified way inside the organism (mostly bound to metallothioneins), and (2) by excreting them but with no changes in concentrations, since excretion and metal uptake rates tended to balance out. According to this author, the excretion of non-essential metals occurs when there is an excess of the detoxified metal in a body compartment. Despite the fact that both the hepatopancreas and gills are directly involved in metal uptake, storage, and excretion due to their great involvement in metallothionein synthesis [64], other authors have reported significantly higher metallothionein concentrations in hepatopancreas than in gills [65], which could explain why higher Pb concentrations were found in the hepatopancreas of crabs fed on mussels from Portmán Bay. Thus, the hepatopancreas of crabs from this group could act as temporary Pb deposits, at least in the short term.

It is important to point out that muscle tissue had the lowest Pb concentrations and was the only tissue that showed significant differences from the other tissues for all treatments. This result confirms our predictions and supports the idea that muscles have a low accumulative potential since they are not metabolically active tissues. Comparable findings have been reported by Bordon et al. [49], who used similar Pb concentrations in *Callinectes* spp., and for all treatments (contaminated food, water, and combined), these authors obtained the lowest amounts of Pb in muscles. Hence, it seems that muscles are not a target tissue for this metal and so Pb accumulation may only begin when an excess reaches muscles via the hemolymph from gills and the hepatopancreas. Despite this typical pattern of low heavy metal accumulation in muscles, there are exceptions in which higher levels are found in muscles than in the hepatopancreas and gills, as has been noted by Mohamed and Osman [66] for *Oreochromis nilotius*. On the other hand, muscle meat from appendages and abdomens are important tissues for pollution control due to their capacity to transfer metals through the food web chain [67] and highlight potential risks to human health. *Callinectes sapidus* is widely consumed due to its protein content (14–19%) and body size, which has led scientists to declare it to be an interesting source of protein for human exploitation [32,68]. Metal concentrations were lower than the maximum permissible levels established by the EU Commission Regulation 2015/1005 [69] for muscle meat in crustaceans (0.5 mg kg^−1^). In this study, the highest Pb concentrations in muscles were obtained from a crab fed on mussels from Portmán Bay (0.261 mg kg^−1^) and exposed to MPs in the water, and so a priori, there would seem to be no reason not to consume meat from those specimens.

No statistical differences in Pb tissue concentrations were found between crabs exposed and not exposed to MPs (Table 1), although the highest Pb concentrations were found in tissues of crabs fed on mussels from Portmán Bay that were exposed to MPs. Based on our results, it seems that an increase in Pb accumulation could occur when MPs are present in the water. When MPs occur in their oxidised form, they can adsorb and act as vectors of contaminants such as heavy metals that are present in the water, thereby increasing the exposure of organisms to metal pollutants [70,71,72]. In the present study, the MPs (AQUATEX 100) were an oxidised polyethylene that could have adsorbed and concentrated the Pb present in the water, which could have facilitated the entrance of Pb bound to MPs in crabs in two ways: (1) in the hepatopancreas while feeding and (2) through the gills in the water. Nevertheless, and as mentioned above, the Pb concentrations in the water were very low, and the exposure time was only seven days. Differences in metal adsorption by virgin and beached polyethylene pellets have been recorded in experiments under estuarine and marine conditions by Holmes et al. [73,74]. According to these authors, the metal uptake by plastics pellets was greater in plastics that had spent more time in seawater (‘aged plastics’) than in others that had not been in contact with water. This difference in metal adsorption is caused presumably by the changes in the surface properties that occur when plastic pellets are in contact with the marine environment. In addition, the adsorption of metal ions can be increased through processes such as photo-oxidative weathering, the accumulation of biofilms and chemical hydrogenous precipitates [73,75,76]. In this sense, Brennecke et al. [77] have reported an increase in heavy metals in MPs after 14 days. In our study, the exposure to MPs was only for seven days and so further studies are necessary to test whether or not this tendency becomes significant as the exposure time increases. In addition, there is concern about the ingestion of MPs. Redondo-Hasselerharm et al. [78] report the presence of MPs in the body and faeces of *Gammarus pulex*, while other studies have concluded that sediments can constitute a source for ingestion of MPs [79]. However, a recent study has provided evidence that, owing to their scavenging and digestive activity, benthic crustaceans could facilitate the fragmentation of the MPs that have accumulated in sediments and thus give rise to a new kind of ‘secondary’ MPs [80]. According to Renzi et al. [81], this process could affect aquatic organisms and contribute to the bioaccumulation of metals and chemical substances released from ingested MPs. Thus, more studies on the interaction of crustaceans with MPs are required to clarify these ideas.

## 4. Conclusions

In conclusion, *Callinectes sapidus* could be used as a bioindicator for studying the occurrence of this non-essential metal (lead) in polluted areas as it does not play any role in crustaceans’ life cycles and its presence permits us to determine how polluted an ecosystem is. The hepatopancreas is the best tissue for measuring Pb concentrations after dietary exposure. The gills may be of interest in highly contaminated zones. Muscles, on the other hand, provide little information relating to environmental conditions and the presence of Pb. The presence of MPs in the water could increase the accumulation of Pb in the tissues of *C. sapidus*. However, to confirm this hypothesis, longer-term studies are still required. Finally, the human consumption of muscle tissue from *C. sapidus* from zones with high Pb content does not seem to constitute a risk for consumers, although there could be some risk if all soft tissues are ingested.

## Figures and Tables

**Table 1:** Descriptive statistics (geometric mean, standard error, minimum and maximum) of the Pb concentrations in tissues (μg g^−1^, ww) and faeces (μg g^−1^, dw) of *C. sapidus* after the 7-day treatment.

Exposure Group: Mussel Origin and MP Exposure	Muscle	Hepatopancreas	Gills	Faeces
San Pedro del Pinatar (without MPs)	0.004 ± 0.006 ^AB^ (*nd*−0.037)	0.065 ± 0.050 ^ab A^ (*nd*−0.322)	0.234 ± 0.072 ^B^ (0.117–0.583)	30.493 ± 4.939 ^a,b^ (18.234–45.046)
San Pedro del Pinatar + MPs	0.017 ± 0.013 ^AB^ (*nd*−0.076)	0.311 ± 0.060 ^cdA^ (0.144–0.588)	0.182 ± 0.390 ^B^ (*nd*−2.178)	34.498 ± 11.447 ^c,d^ (16.974–55.806)
Portmán Bay (without MPs)	0.003 ± 0.026 ^AB^ (*nd*−0.127)	1.178 ± 0.158 ^aceAC^ (0.876–1.884)	0.384 ± 0.199 ^BC^ (0.063–1.406)	680.132 ± 46.766 ^a,c^ (550.439–821.053)
Portmán Bay + MPs	0.008 ± 0.044 ^AB^ (*nd*−0.261)	1.565 ± 0.455 ^bdfAC^ (0.511–3.598)	0.519 ± 0.060 ^BC^ (0.374–0.758)	649.338 ± 56.101 ^b,d^ (503.079–772.040)
Segura River, ‘Zero Hour’	0.006 ± 0.018 ^AB^ (*nd*−0.098)	0.174 ± 0.020 ^ef A^ (0.106–0.257)	0.095 ± 0.045 ^B^ (0.004–0.297)	n/a

For each tissue and faeces: exposure groups with the same lowercase had significant statistical differences. For each exposure group: tissues (muscle, hepatopancreas, and gills) with the same capital letter had significant statistical differences. MPs=microplastics; n/a = not analysed; *nd* = not detected.

## References

[1] Hutchinson T.H., Lyons B.P., Thain J.E., Law R.J. (2013). Evaluating legacy contaminants and emerging chemicals in marine environments using adverse outcome pathways and biological effects-directed analysis. Mar. Pollut. Bull..

[2] Martellini T., Guerranti C., Scopetani C., Ugolini A., Chelazzi D., Cincinelli A. (2018). A snapshot of microplastics in the coastal areas of the Mediterranean Sea. Trends Anal. Chem..

[3] Llorca M., Álvarez-Muñoz D., Ábalos M., Rodríguez-Moraz S., Santos L.H.M.L.M., León V.M., Campillo J.A., Martínez-Gómez C., Abad E., Farré M. (2020). Microplastics in Mediterranean coastal area: Toxicity and impact for the environment and human health. Trends Anal. Chem..

[4] Raza M., Hussain F., Lee J.-Y., Shakoor M.B., Kwon K.D. (2017). Groundwater status in Pakistan: A review of contamination, health risks, and potential needs. Crit. Rev. Environ. Sci. Technol..

[5] Torres P., Rodrigues A., Prestes A.C.L., Neto A.I., Álvaro N., Martins G.M. (2020). The Azorean edible abalone *Haliotis tuberculata*, an alternative heavy metal-free marine resource?. Chemosphere.

[6] Franco-Solórzano J.G. (2015). Determinación de niveles de mercurio, cadmio, níquel, cromo y plomo en tejido blando, hepatopáncreas en la jaiba azul (Callinectes arcuatus) y sedimento en los ramales del estero salado.

[7] WHO (2021). Exposure to Lead: A Major Public Health Concern.

[8] US EPA (2021). List of Priority Pollutants. United States Environmental Protection Agency. Priority Pollutant List. Washington, USA. https://www.epa.gov/sites/production/files/2015-09/documents/priority-pollutant-list-epa.pdf.

[9] (2015). Real Decreto 817/2015, de 11 de septiembre, por el que se establecen los criterios de seguimiento y evaluación del estado de las aguas superficiales y las normas de calidad ambiental. Boletín Oficial del Estado.

[10] Sánchez-Marín P. (2010). Dissolved Organic Matter and Pb and Cu Bioavailability in the Marine Environment. A Test of Free Ion Based Models. Ph.D. Thesis.

[11] Mallongi A., Ane R.L., Birawida A.B. (2017). Ecological Risks of Contaminated Lead and the Potential Health Risks among School Children in Makassar Coastal Area, Indonesia. J. Environ. Sci. Technol..

[12] PlasticsEurope (2021). Plastics—The Facts 2019. An Analysis of European Plastics Production, Demand and Waste Data. Brussels, Belgium. https://www.plasticseurope.org/application/files/9715/7129/9584/FINAL_web_version_Plastics_the_facts2019_14102019.pdf.

[13] Jambeck J.R., Geyer R., Wilcox C., Siegler T.R., Perryman M., Andrady A., Narayan R., Law K.L. (2015). Plastic waste inputs from land into the ocean. Science.

[14] Andrady A.L. (2017). The plastic in microplastics: A review. Mar. Pollut. Bull..

[15] Alimi O.S., Budarz J.F., Hernandez M.L., Tufenkji N. (2018). Microplastics and nanoplastics in aquatic environments: Aggregation, deposition, and enhanced contaminant transport. Environ. Sci. Technol..

[16] Lusher A.L., Hernández-Milian G., O’Brien J., Berrow S., O’Connor I., Officer R. (2015). Microplastic and macroplastic ingestion by a deep diving, oceanic cetacean: The True’s beaked whale *Mesoplodon mirus*. Environ. Pollut..

[17] Allen S., Allen D., Phoenix V.R., Le Roux G., Jiménez P.D., Simonneau A., Binet S., Galop D. (2019). Atmospheric transport and deposition of microplastics in a remote mountain catchment. Nat. Geosci..

[18] Ye S., Cheng M., Zeng G., Tan X., Wu H., Liang J., Shen M., Song B., Liu J., Yang H. (2020). Insights into catalytic removal and separation of attached metals from natural-aged microplastics by magnetic biochar activating oxidation process. Water Res..

[19] Fossi M.C., Pedà C., Compa M., Tsangaris C., Alomar C., Claro F., Ioakeimidis C., Galgani F., Hema T., Deudero S. (2018). Bioindicators for monitoring marine litter ingestion and its impact on Mediterranean biodiversity. Environ. Pollut..

[20] Genç T.O., Yilmaz F. (2017). Metal Accumulations in Water, Sediment, Crab (*Callinectes sapidus*) and Two Fish Species (*Mugil cephalus* and *Anguilla anguilla*) from the Köyceğiz Lagoon System–Turkey: An Index Analysis Approach. Bull. Environ. Contam. Toxicol..

[21] Fernández B., Albentosa M. (2019). Dynamic of small polyethylene microplastics (≤10 μm) in mussel’s tissues. Mar. Pollut. Bull..

[22] Salvat-Leal I., Verdiell D., Parrondo P., Barcala E., Romero D. (2020). Assessing lead and cadmium pollution at the mouth of the river Segura (SE Spain) using the invasive blue crab (*Callinectes sapidus* Rathbun, 1896, Crustacea, Decapoda, Portunidae) as a bioindicator organism. Reg. Stud. Mar. Sci..

[23] Williams A.B. (1984). Shrimps, Lobsters, and Crabs of the Atlantic Coast of the Eastern United States, Maine to Florida.

[24] Reichmuth J.M., Weis P., Weris J.S. (2010). Bioaccumulation and depuration of metals in blue crabs (*Callinectes sapidus* Rathbun) from a contaminated and clean estuary. Environ. Pollut..

[25] Millikin M.R., Williams A.B. (1984). Synopsis of Biological Data on the Blue Crab, Callinectes Sapidus Rathbun.

[26] Williams H.A., Coen L.D., Stoelting M.S. (1990). Seasonal abundance, distribution and habitat selection of jovenes *Callinectes sapidus* (Rathbun) in the Northern Gulf of Mexico. J. Exp. Mar. Biol. Ecol..

[27] Rosas V.C., Sánchez A., De la Lanza G., Cáceres C. (1994). Fisiología de la adaptación de los crustáceos decápodos al ambiente lagunar estuarino. Lagunas Costeras y el Litoral Mexicano.

[28] Lahera C.S., Betanzos-Vega A., Hurtado E.G., Cruz Y.M. (2016). Influencia de la temperatura y salinidad en la distribución espacial y temporal de jaiba azul (*Callinectes sapidus*, Rathbun, 1896) en bahía de Buenavista, Cuba. Rev. Cuba. Investig. Pesq..

[29] Garcia L., Pinya S., Colomar V., París T., Puig M., Rebassa M., Mayol J. (2018). The first recorded occurrences of the invasive crab Callinectes sapidus Rathbun, 1896 (Crustacea: Decapoda: Portunidae) in coastal lagoons of the Balearic Islands (Spain). BioInvasions Rec..

[30] Küçükgülmez A., Çelik M. (2008). Aminoacid composition of blue crab (*Callinectes sapidus*) from the North Eastern Mediterranean Sea. J. Appl. Environ. Biol. Sci..

[31] Zotti M., Del Coco L.D., De Pascali S.A., Migoni D., Vizzini S., Mancinelli G., Fanizzi F.P. (2016). Comparative analysis of the proximate and elemental composition of the blue crab *Callinectes sapidus*, the warty crab *Eriphia verrucosa*, and the edible crab Cancer pagurus. Heliyon.

[32] Mancinelli G., Chainho P., Cilenti L., Falco S., Kapiris K., Katselis G., Ribeiro F. (2017). The Atlantic blue crab *Callinectes sapidus* in southern European coastal waters: Distribution, impact and prospective invasion management strategies. Mar. Pollut. Bull..

[33] Adams D.H., Engel M.E. (2014). Mercury, lead, and cadmium in blue crabs, *Callinectes sapidus*, from the Atlantic coast of Florida, USA: A multipredator approach. Ecotoxicol. Environ. Saf..

[34] Box A., Sureda A., Galgani F., Pons A., Deudero S. (2007). Assessment of environmental pollution at Balearic Islands applying oxidative stress biomarkers in the mussel *Mytilus galloprovincialis*. Biochem. Physiol. Part C Toxicol. Pharmacol..

[35] Höher N., Turja R., Brenner M., Nyholm J.R., Östin A., Leffler P., Butrimavičienė L., Baršiene J., Halme M., Karjalainen M. (2019). Toxic effects of chemical warfare agent mixtures on the mussel *Mytilus trossulus* in the Baltic Sea: A laboratory exposure study. Mar. Environ. Res..

[36] Maser E., Strehse J.S. (2020). “Don’t Blast”: Blast-in-place (BiP) operations of dumped World War munitions in the oceans significantly increase hazards to the environment and the human seafood consumer. Arch. Toxicol..

[37] Schuster R., Strehse J.S., Ahvo A., Turja R., Maser E., Bickmeyer U., Lehtonen K.K., Brenner M. (2020). Exposure to dissolved TNT causes multilevel biological effects in Baltic mussels (*Mytilus* spp.). Mar. Pollut. Bull..

[38] García-Navarro J.A., Franco L., Romero D. (2017). Differences in the accumulation and tissue distribution of Pb, Cd, and Cu in Mediterranean mussels (*Mytilus galloprovincialis*) exposed to single, binary, and ternary metal mixtures. Environ. Sci. Pollut. Res..

[39] Li J., Lusher A.L., Rotchell J.M., Deudero D., Turra A., Bråte I.L.N., Sun C., Hossain M.S., Li Q., Kolandhasamy P. (2019). Using mussel as a global bioindicator of coastal microplastic pollution. Environ. Pollut..

[40] Benedicto J., Martínez-Gómez C., Guerrero J., Jornet A., Rodríguez C. (2008). Metal contamination in Portman Bay (Murcia, SE Spain) 15 years after the cessation of mining activities. Cienc. Mar..

[41] Pichaud N., Pellerin J., Fournier M., Gauthier-Clerc S., Rioux P., Pelletier É. (2008). Oxidative stress and immunologic responses following a dietary exposure to PAHs in Mya arenaria. Chem. Cent. J..

[42] Phillips D.J.H. (1976). The common mussel Mytilus edulis as an indicator of pollution by zinc, cadmium, lead and copper. I. Effects of environmental variables on uptake of metals. Mar. Biol..

[43] Martínez-Sánchez M.J., Pérez-Sirvent C., Garcia-Lorenzo M.L., Martínez-Lopez S., Bech J., Hernández C., Martínez L.B., Molina J., Bech J., Bini C., Pashkevich M.A. (2017). Ecoefficient in situ technologies for the remediation of sites affected by old mining activities: The case of Portman Bay. Assessment, Restoration and Reclamation of Mining Influenced Soils.

[44] Benavente D., Pla C., Valdes-Abellan J., Cremades-Alted S. (2020). Remediation by waste marble powder and lime of jarosite-rich sediments from Portman Bay (Spain). Environ. Pollut..

[45] Serrano R., Gras L., Giménez-Casalduero F., del-Pilar-Ruso Y., Grindlay G., Mora J. (2019). The role of *Cymodocea nodosa* on the dynamics of trace elements in different marine environmental compartments at the Mar Menor Lagoon (Spain). Mar. Pollut. Bull..

[46] Green D.S., Boots B., O’Connor N.E., Thompson R. (2017). Microplastics Affect the Ecological Functioning of an Important Biogenic Habitat. Environ. Sci. Technol..

[47] (1995). Reliable Evaluation of Low-Level Contamination of Food–Workshop in the Frame of GEMS/Food-EURO.

[48] Fernández B., Campillo J.A., Martínez-Gómez C., Benedicto J. (2010). Antioxidant responses in gills of mussel (*Mytilus galloprovincialis*) as biomarkers of environmental stress along the Spanish Mediterranean coast. Aquat. Toxicol..

[49] Bordon I.C., Emerenciano A.K., Melo J.R.C., Silva J.R.M.C.D., Favaro D.I.T., Gusso-Choueri P.K., Campos B.G., Abessa D.M.S. (2018). Implications on the Pb bioaccumulation and metallothionein levels due to dietary and waterborne exposures: The *Callinectes danae* case. Ecotoxicol. Environ. Saf..

[50] Türkmen A., Türkmen M., Tepe Y., Mazlum Y., Oymael S. (2006). Metal concentrations in blue crab (*Callinectes sapidus*) and mullet (*Mugil cephalus*) in Iskenderun Bay, Northern East Mediterranean, Turkey. Environ. Contam. Toxicol..

[51] Ayas D., Ozogul Y. (2011). The effects of sex and seasonality on the metal levels of different muscle tissues of mature Atlantic blue crabs (*Callinectes sapidus*) in Mersin Bay, north-eastern mediterranean. Int. J. Food Sci. Technol..

[52] Çoğun H.Y., Firat Ö., Aytekin T., Firidin G., Varkal H., Temiz Ö., Kargın F. (2017). Heavy Metals in Blue Crab (*Callinectes sapidus*) in Mersin Bay, Turkey. Bull. Environ. Contam. Toxicol..

[53] Karouna-Renier N.K., Snyder R.A., Allison J.G., Wagner M.G., Rao R.K. (2007). Accumulation of organic and inorganic contaminants in shellfish collected in estuarine waters near Pensacol, Florida: Contamination profiles and risks to human consumers. Environ. Pollut..

[54] Bordon I.C.A.C., Sarkis J.E.S., Tomás A.R.G., Scalco A., Lima M., Hortellani M.A., Andrade N.P. (2012). Assessment of metal concentration in muscles of blue crab, *Callinectes danae* S., from the Santos Estuarine system. Bull. Environ. Contam. Toxicol..

[55] Lavradas R.T., Hauser-Davis R.A., Lavandier R.C., Rocha R.C., Saint’ Pierre T.D., Seixas T., Kehrig H.A., Moreira I. (2014). Metal, metallothionein and glutathione levels in blue crab (*Callinectes* sp.) specimens from southeastern Brazil. Ecotoxicol. Environ. Saf..

[56] Gutiérrez-Peña L.V., Picón D., Gutiérrez L.A., Prada M., Carrero P.E., Delgado-Cayama Y.J., Gutiérrez E.O., Morón M., González C.E., Lara N.D. (2018). Heavy metals in soft tissue of blue crab (*Callinectes sapidus*) of Puerto Concha, Colon Municipality, Zulia State. Adv. Biomed..

[57] Duruibe J.O., Ogwuegbu M.O.C., Egwurugwu J.N. (2007). Heavy metal pollution and human biotoxic effects. Int. J. Phys. Sci..

[58] Engel D.W. (1983). The intracellular partitioning of trace metals in marine shellfish. Sci. Total. Environ..

[59] Sivaperumal P., Sankar T.V., Nair P.G. (2007). Heavy metal concentration in fish, shellfish and fish products from internal markets of India vis-avis international standards. Food Chem..

[60] Legras S., Mouneyrac C., Amiard J.C., Triquet-Amiard C., Rainbow P. (2000). Changes in metallothionein concentrations in response to variation in natural factors (salinity, sex, weight) and metal concentration in crabs from a metal-rich estuary. J. Exp. Mar. Biol. Ecol..

[61] Vázquez F.J. (2005). Toxicidad Comparada de Zinc, Plomo y Mercurio para Zoea I de *Chasmagnathus granulatus* (Brachyura). Bachelor’s Thesis.

[62] Bordon I.C.A.C., Sarkis J.E., Andrade N.P., Hortellani M.A., Favaro D.I., Kakazu M.H., Cotrim M.E., Lavradas R.T., Moreira I., Saint’Pierre T.D. (2016). An environmental forensic approach for tropical estuaries based on metal bioaccumulation in tissues of *Callinectes danae*. Ecotoxicology.

[63] Rainbow P.S. (2002). Trace metal concentrations in aquatic invertebrates: Why and so what?. Environ. Pollut..

[64] Amiard J.C., Amiard-Triquet C., Barka S., Pellerin J., Rainbow P.S. (2006). Metallothioneins in aquatic invertebrates: Their role in metal detoxification and their use as biomarkers. Aquat. Toxicol..

[65] Martins C.D.M.G., Barcarolli I.F., Menezes E.J., Giacomin M.M., Wood C.M., Bianchini A. (2009). Acute toxicity, accumulation and tissue distribution of copper in the blue crab *Callinectes sapidus* acclimated to different salinities: In vivo and in vitro studies. Aquat. Toxicol..

[66] Mohamed E.H.A., Osman A. (2014). Heavy metals concentration in water, muscles and gills of *Oreochromis niloticus* collected from the sewage-treated water and the White Nile. Aquac. Int..

[67] Tunçsoy M., Erdem C. (2014). Accumulation of copper, zinc and cadmium in liver, gill and muscle tissues of *Oreochromis niltoticus* exposed to these metals separately and in mixture. Fresenius Environ. Bull..

[68] Martínez M.A., Velázquez G., Cando D., Núñez-Flores R., Borderías A.J., Moreno H.M. (2017). Effects of high pressure processing on protein fractions of blue crab (*Callinectes sapidus*) meat. Emerg. Technol..

[69] (2015). Commission Regulation (EU) 2015/1005 of 25 June 2015 Amending Regulation (EC) N° 1881/2006 as Regards Maximum Levels of Lead in Certain Foodstuffs.

[70] Reisser J., Shaw J., Hallegraeff G., Proietti M., Barnes D.K.A., Thums M., Wilcox C., Hardesty B.D., Pattiaratchi C. (2014). Millimeter-Sized Marine Plastics: A New Pelagic Habitat for Microorganisms and Invertebrates. PLoS ONE.

[71] Hodson M.E., Duffus-Hodson C.A., Clark A., Prendergast-Miller M.T., Thorpe K.L. (2017). Plastic bag derived-microplastics as a vector for metal exposure in terrestrial invertebrates. Environ. Sci. Technol..

[72] Massos A., Turner A. (2017). Cadmium, lead and bromine in beached microplastics. Environ. Pollut..

[73] Holmes L.A., Turner A., Thompson R.C. (2012). Adsorption of trace metals to plastic resin pellets in the marine environment. Environ. Pollut..

[74] Holmes L.A., Turner A., Thompson R.C. (2014). Interactions between trace metals and plastic production pellets under estuarine conditions. Mar. Chem..

[75] Mato Y., Isobe T., Takada H., Kanehiro H., Ohtake C., Kaminuma T. (2001). Plastic resin pellets as a transport medium for toxic chemicals in the marine environment. Environ. Sci. Technol..

[76] Morét-Ferguson S., Law K.L., Proskurowski G., Murphy E.K., Peacock E.E., Reddy C.M. (2010). The size, mass, and composition of plastic debris in the western North Atlantic Ocean. Mar. Pollut. Bull..

[77] Brennecke D., Duarte B., Paiva F., Caçador I., Canning-Clode J. (2016). Microplastics as vector for heavy metal contamination from the marine environment. Estuar. Coast. Shelf Sci..

[78] Redondo-Hasselerharm P.E., Falahudin D., Peeters E.T.H.M., Koelmans A.A. (2018). Microplastics effect thresholds for freshwater benthic macroinvertebrates. Environ. Sci. Technol..

[79] Thompson R.C., Olsen Y., Mitchell R.P., Davis A., Rowland S.J., John A.W.G., McGonigle D., Russell A.E. (2004). Lost at sea: Where is all the plastic?. Science.

[80] Cau A., Avio C.G., Dessi C., Moccia D., Pusceddu A., Regoli F., Cannas R., Follesa M.C. (2020). Benthic crustacean digestion can modulate the environmental fate of microplastics in the deep sea. Environ. Sci. Technol..

[81] Renzi M., Cilenti L., Scirocco T., Grazioli E., Anselmi S., Broccoli A., Pauna V., Provenza F., Specchiulli A. (2020). Litter alien species of possible commercial interest: The blue crab (*Callinectes sapidus* Rathbun, 1896) as case study. Mar. Pollut. Bull..

